# Investigation of the Dynamism of Nanosized SOA Particle Formation in Indoor Air by a Scanning Mobility Particle Sizer and Proton-Transfer-Reaction Mass Spectrometry

**DOI:** 10.3390/molecules25092202

**Published:** 2020-05-08

**Authors:** Klaudia Pytel, Renata Marcinkowska, Bożena Zabiegała

**Affiliations:** Department of Analytical Chemistry, Faculty of Chemistry, Gdańsk University of Technology, 11/12 Narutowicza Str. 80-233 Gdańsk, Poland; klapytel@student.pg.edu.pl (K.P.); bozena.zabiegala@pg.edu.pl (B.Z.)

**Keywords:** monoterpenes, secondary organic aerosol, indoor air quality, PTR-TOF-MS, SMPS, real-time measurement techniques

## Abstract

Terpenes are VOCs of particular importance, since they are emitted from a wide range of indoor sources and are considered to be precursors of Secondary Organic Aerosol (SOA) formation. It has been proven that SOA particles, especially nanosized ones, pose a threat to human health. In this research, experiments with the application of an environmental chamber and real-time measurement techniques were carried out to investigate in a complimentary way the formation of monoterpene oxidation products and nanosized SOA particles initiated by monoterpene ozonolysis. Proton-Transfer-Reaction Mass Spectrometry with a Time-Of-Flight analyzer (PTR-TOF-MS) and a Scanning Mobility Particle Sizer (SMPS) were applied to determine in real time the dynamism of the formation of the corresponding terpene ozonolysis products and submicron SOA particles. Results proved that firstly, oxidation products were formed, and then, they underwent nucleation and condensation, forming particles whose diameters grew with time. The oxidation products formed were different depending on the type of terpenes applied. The comparison of the results obtained during the experiments with gaseous standard mixtures and real samples commonly present and used in indoor air revealed that the diversified chemical composition of the emission source had implications for both the particle formation initiated by the oxidation of essential oil components and the chemical reactions occurring via the oxidation process. With the instrumentation utilized, the concentration changes at the level of a few ppbv could be monitored.

## 1. Introduction

The fact that people spend only about 15% of their time outdoors [1,2,3] makes indoor air quality an important factor influencing human health and wellbeing. Volatile Organic Compounds (VOCs) are chemicals most commonly influencing indoor air chemistry [4]. Broadened research aimed to seek chemicals contributing to poor indoor air quality revealed that terpenes are one of the groups of VOCs strongly influencing indoor air chemistry and quality. Due to the wide range of emission sources (the main ones are: cleaning agents, air fresheners, wooden furniture, cosmetic products [5,6]), it is almost impossible to avoid or get rid of their presence in enclosed spaces. The issue concerning terpenes is associated with their high reactivity. Single or multiple double bonds in terpenes’ structure are responsible for extremely fast reactions between terpenes and oxidants (ozone, hydroxyl radicals, nitrate radicals) present in indoor air [7]. Currently, scientists are sure of the first few steps of terpenes’ reactions. It has been proven that firstly, terpene is attacked by ozone in the place of double bonding, and a primary ozonide (PO) is formed. PO is then decomposed to form the so-called “Criegee intermediate”, which is highly reactive and will undergo further reactions, which are still under investigation [8,9,10,11,12]. Some terpenes’ oxidation products may remain in a gaseous phase, while others may undergo homogeneous nucleation or condensation on preexisting particles. These processes result in the formation of ultrafine secondary organic aerosol (SOA) particles, which further evolve in size towards larger diameters [13]. An important factor influencing indoor particle growth is the presence of seed particles. Seed particles are present in indoor air mainly from the outdoor sources. A high concentration of seed particles in indoor air increases the partitioning of semi-volatile oxidation products, which leads to increased particle mass concentration indoors [14].

Investigation of the particle size distribution in indoor air is of great importance, since the particle size determines where it would deposit in the respiratory track [15]. There are several mechanisms responsible for particle deposition in a given part of the human respiratory track. Mainly, this is by diffusion, sedimentation, and impaction. Deposition mechanisms are related to particle sizes. Hence, small particles with diameters less than 0.5 µm are deposited mainly through diffusion, which takes place in the alveolar interstitium, deep in the respiratory track. Particles of a diameter greater than 0.5 µm are mainly deposited by sedimentation in the alveolar interstitium and tracheobronchial airway. Particles greater than 1 µm are most commonly deposited by impaction in the tracheobronchial airway [16,17,18]. SOA particles have submicron diameters, which allow them to penetrate the human respiratory track deep into pulmonary alveoli and further into the bloodstream [19,20]. Numerous in vitro, in vivo, and sensory studies have demonstrated the irritating and inflammatory properties of SOA particles created by terpenes’ oxidation. According to the literature data, SOA particles are responsible for: initiation of the anti-inflammatory response in human alveolar epithelium cells [21], a breath frequency decrease in mice (which is a sign of respiratory track irritation), histopathological changes in lung cells [22,23,24,25], and finally, an increase in eye blink frequency in humans (which is a sign of eye irritation) [26,27]. Due to dynamic changes in terpenes’ indoor chemistry, it is still challenging to determine the chemical composition of the created SOA. Many model and experimental studies were carried out in order to determine the mechanism of terpenes’ reactions with various indoor oxidants. The prediction of the reaction products and their further transformations is challenging, because terpenes’ chemistry strongly depends on many factors such as humidity and the availability of substrates [28,29]. In most of the studies, reactions involving ozone were investigated because of its abundance in indoor air; however, research concerning terpenes’ reactions with the hydroxyl radical is also frequently carried out [30,31]. Mathematical modelling is highly useful in predicting the products of terpene oxidation reactions. For example, the DFT method was successfully applied to predict the products of d-limonene ozonolysis. The model created predicted the formation of keto-limonene, limonoic acid, and 7-hydroxylimononaldehyde or 3-isopropenyl-6-oxo-7-hydroxy-hetanal [32], which coincided with laboratory studies. However, laboratory research revealed even more oxidation products such as limononaldehyde and keto-limononaldehyde [33]. Until now, numerous studies have been carried out to determine terpenes’ oxidation mechanisms, and all of them proved that there was a wide range of different products created due to the reactions of terpenes with different oxidants and in different environmental conditions [34,35,36,37,38]. Terpenes’ oxidation products are noticeably contributing to indoor air quality decrease by the formation of SOA, which was confirmed also by sensory analysis [39].

Taking into account the numbers of published papers dealing with terpene-derived SOA so far, it may be stated that studies on outdoor atmospheric chemistry significantly outnumber those related to indoor aerosol particles’ formation (only approximately 10% of those published in the past five years addressing SOA formation concerned indoor air). However, a point to be made here is that indoor gas-phase chemistry has been thoroughly investigated for the past 20 years, which resulted in numerous papers addressing SOA formation via ozone/terpenoid reactions. As directions for future research, the indoor chemistry community indicates the application of fast-time-response instrumentation with low limits of detection in field campaigns, which would allow evaluating the results of real sample/environment studies with regard to model (chamber) studies [40]. Studies already reported in the literature addressing “real-world” products most of all deal with personal care or household products (e.g., air fresheners, degreasers, cleaners, perfumes etc.) [41,42].

The aim of this study was to investigate the reactions of terpenes (both generated as a standard gaseous mixture and emitted from products commonly present indoors) with ozone as an oxidant and their influence on SOA formation, growth, and distribution in time. In this work, particle number density was measured, since it brings valuable information with regard to assessment of the risk of SOA ultrafine particles’ formation in indoor air on human health [43]. Mass concentration is recognized as not useful to describe the phenomenon of particle interactions with membranes or gills like sorption, translocation, or localized chemical exposure, because the same mass of smaller particles would penetrate biological barriers, when larger particles will not [43,44,45,46]. By the application of Proton-Transfer-Reaction Mass Spectrometry (PTR-MS) and a Scanning Mobility Particle Sizer (SMPS), it was possible to monitor changes in the concentration of trace or even ultra-trace organic constituents over very short periods of time with high sensitivity and ultrafine particle formation, at the same time obtaining detailed particle size distribution changes with time, both initiated by the ozonolysis of reactive VOCs commonly present in indoor air. Studies on the potential impact of terpenes’/ozone-initiated chemistry on indoor air quality involving constituents of products commonly present and used indoors are of great importance since they might contribute to solving the issue of adverse health effects that occupants report after overlong indoor residence.

## 2. Results and Discussion

**Figure 1:**
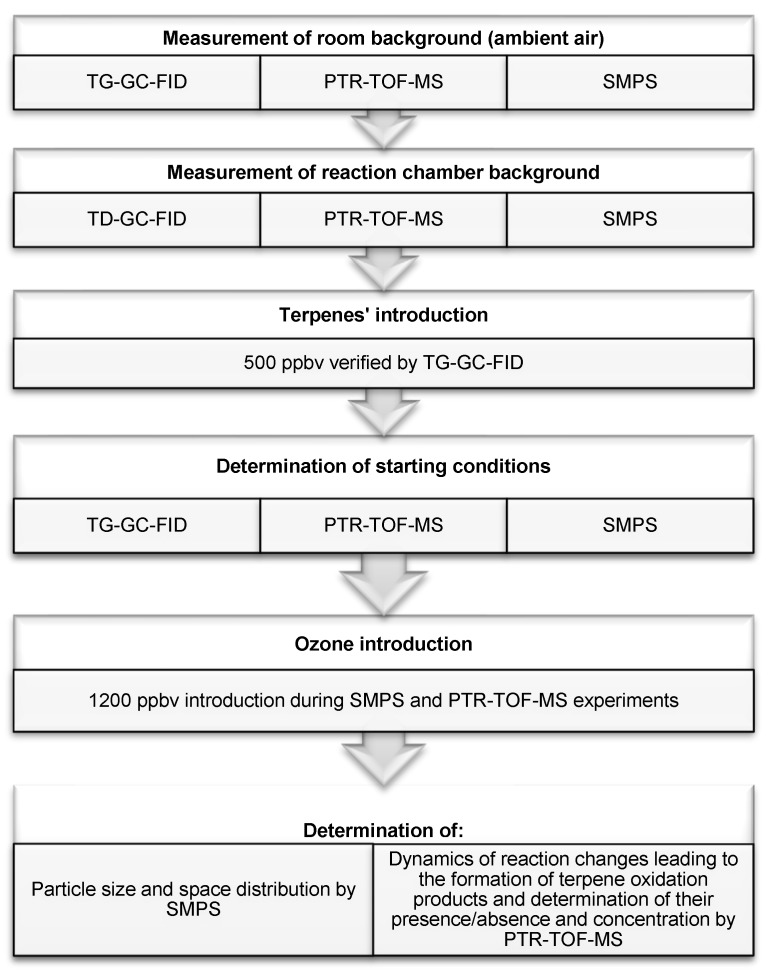
The main steps of the experimental part.

The general scheme of the procedure repeated in each experiment and details regarding ozone and terpenes’ concentrations are presented in Figure 1. The concentrations of reagents subjected to ozonolysis reaction were set empirically, based on the results of preliminary experiments, taking into consideration the repeatability of measurements and the clarity of the results. Each of the experiments carried out was repeated at least three times, and the spread of the results did not exceed 20%. Measurement time was set with reference to a typical residence time of indoor air (the order of 1–2 h [47]), which governed the likelihood that given gaseous and particle constituent-related processes would occur.

### 2.1. Experiments with Gaseous Standard Mixtures

The first part of this study involved the investigation of the dynamism of the formation and chemistry of nanosized SOA particles created via the ozonolysis reaction of monoterpenes introduced into the reaction chamber as gaseous standard mixtures (see the Materials and Methods section for details).

#### 2.1.1. SMPS Measurements

The results obtained by application of SMPS are presented in the form of graphs showing the change of the particle size distribution with time. Particle density was defined as the particle number density per cubic centimeter (particles/cm^3^), while one measurement cycle was equal to 90 s. The particle number density unit was applied, because the mass of nanoparticles was negligible (therefore almost impossible to measure) and the particle mass concentration would not provide any relevant information about the change of particle distribution in time. The particle size distribution will be denoted and abbreviated as “particle distribution” or “PSD”, while particle number density or concentration will be denoted as “particle density” or “PND”. Important information on SMPS experiments discussed in the following text is presented in Table 1.

**Figure 2:**
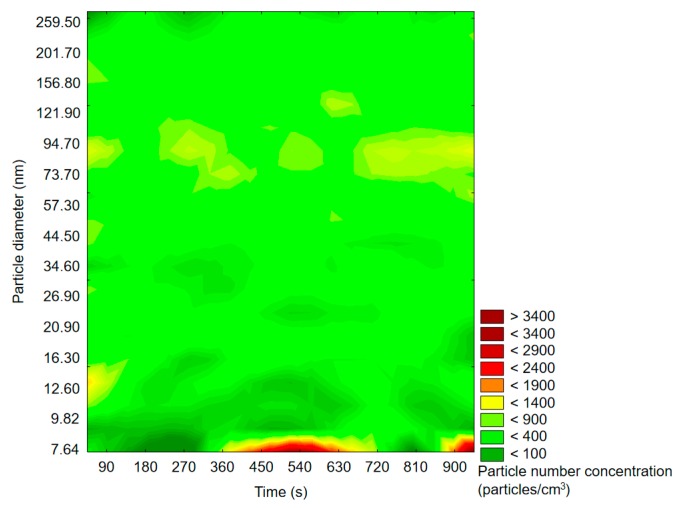
Exemplary results of the SMPS measurement of background particle size and space distribution.

Figure 2 shows exemplary particle measurement of reaction chamber ambient air, without terpenes’ introduction (Step 1 in Figure 1).

The particle size distribution was rather uniform during the whole measurement. An increase in particle number density at the very bottom of the graph indicated that particles of a diameter smaller than 8 nm were possibly abundant, but the applied device settings did not allow monitoring particles of less than 7.64 nm in diameter. Background measurement showed that the magnitude of the particle number concentration before oxidation experiment was negligible taking into consideration the magnitude of PNDs after oxidant introduction; therefore, the possible interference deriving from background air may be excluded.

**Figure 3:**
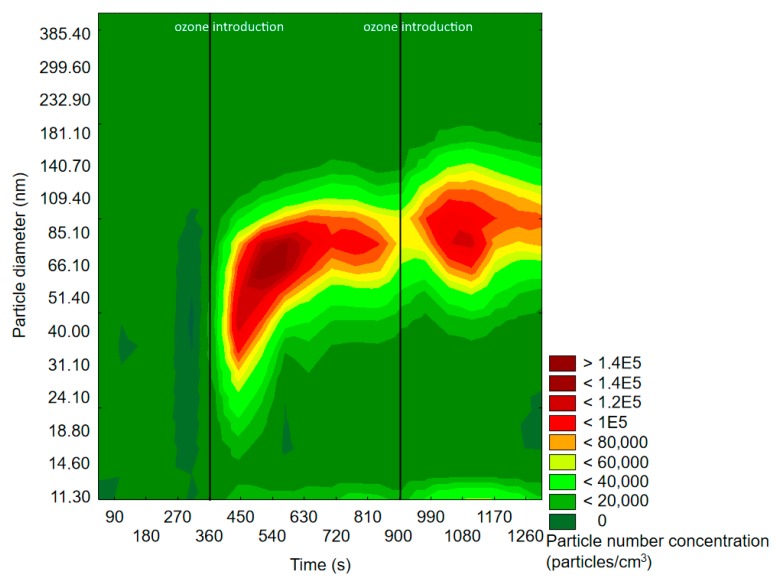
Exemplary results of the SMPS measurement of particle size and space distribution changes initiated by α-pinene and O_3_ reaction.

**Figure 4:**
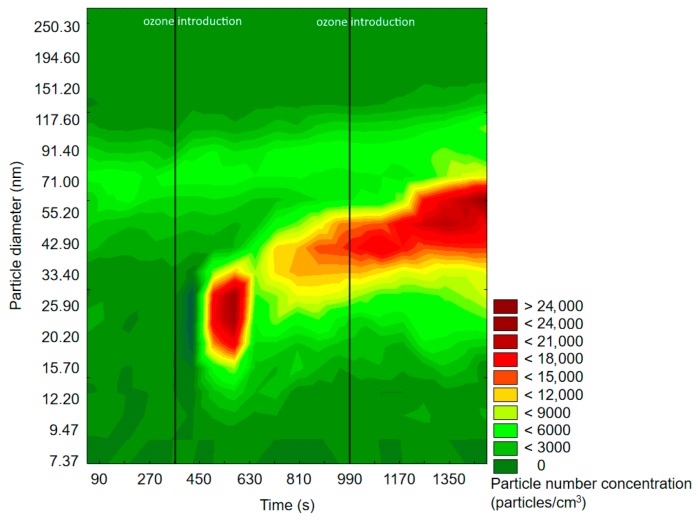
Exemplary results of SMPS measurement of particle size and space distribution changes initiated by d-limonene and O_3_ reaction.

Figure 3 and Figure 4 represent exemplary graphs obtained during the experiments, when either α-pinene or d-limonene (separately) and O_3_ were introduced into the reaction chamber.

Before ozone introduction, dominant particles were of a diameter in the range of 75–100 nm. In Figure 4 (experiment with d-limonene), there is a longitudinal area of increased PSD formed by particles of 55–117 nm, which was present throughout the whole experiment, therefore not related to oxidation processes. In both cases, the effects of ozone introduction were comparable: first, it initiated the burst of fine particles of a diameter <50 nm, which was followed by rapid PND increase and a shift of the maximum concentration towards particles of diameters greater than 60 nm. A similar trend was observable after subsequent ozone introduction. The first ozone introduction induced the formation of new particles, which further nucleated and grew with time into larger particles. Subsequent ozone addition resulted in both homogenous nucleation and coagulation onto already existing particles. In the case of d-limonene oxidation, after the second ozone introduction, there was a constant PND increase by the end of the measurement, which was not observed in the experiment with α-pinene. A visible difference in the course of the formation of ultrafine SOA particles in the case of α-pinene and d-limonene ozonolysis was possibly related to the difference in particle number concentration, which strongly influenced the aerosol condensation sink.

**Figure 5:**
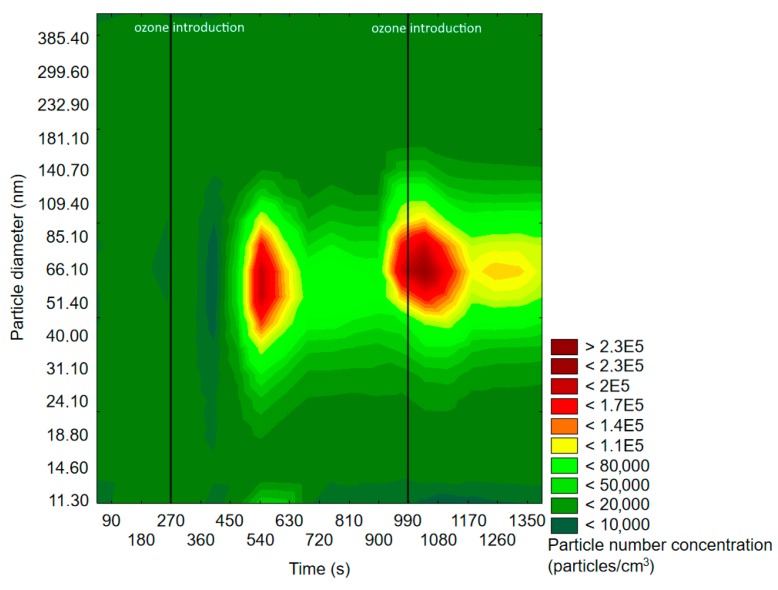
Exemplary results of SMPS measurement of particle size and space distribution changes initiated by α-pinene + d-limonene and O_3_ reaction.

Figure 5 represents exemplary results of the investigation of the particle size distribution initiated via the reaction of the mixture of two terpenes (α-pinene and d-limonene) with ozone.

The observed trend of particle growth was, as expected, similar to those reported in the case of experiments with single monoterpenes: the initial burst of particles of diameters <100 nm was followed by a decrease in PND in a given size range and a subsequent increase in the numbers of particles of a larger size. The PND decrease at the end of the measurement accompanied with constant PSD shift towards particles of bigger diameters was most likely related to the fact that given the magnitude of PND after the second ozone addition into the reaction chamber, enhanced coagulation of newly formed particles with already existing ones took place.

All of the experiments conducted with SMPS clearly indicated the importance of the oxidant’s role in the formation of ultrafine particles that could form SOA. On the basis of the obtained results, one may state that terpenes without oxidant presence did not influence the background particle number density and distribution, but each introduction of an oxidant caused visible changes. For all of the experiments, each introduction of ozone resulted in PND increase. Yet another repeatable trend was that firstly, particles of a smaller diameter were formed, and with the passing of time, the maximum particle number density shifted towards particles of a bigger diameter due to the condensation and nucleation phenomena. Particles of diameters <20 nm were presumably formed in the initial steps of aerosol formation [48]; however, SMPS instrumentation settings did not allow measuring them before the nucleation event. A point to make here is that according to the literature data, the formation of the finest aerosol particles (<5 nm) is assigned to oxidation by OH radicals, whereas ozonolysis plays a greater role when the particles exceeded a diameter of 5 nm [49]. The observed characteristics of ultrafine particles’ formation was analogous to nucleation processes occurring in atmospheric air, even though in this experiment, significantly higher (closer to values reported in indoor air) mixing ratios of monoterpenes and ozone were applied.

Similar phenomena of ultrafine particle formation dynamism were observed also in other reported studies on terpene-induced particle formation in indoor environments [50,51,52]. For instance, in the latter literature example, subsequent limonene introduction caused the same effect as subsequent ozone introduction done in the case of the experiments discussed here, namely initiating another number concentration increase of small particles, followed by size distribution shift, enhancing first the PND increase and distribution pattern. Moreover, the greatest PND increase in the reference study [52] was observed for particles of a diameter between 9 and 50 nm, which was in accordance with the results of the experiments presented in this paper. This proved that initially, small particles were formed, and with the passing of time, the distribution shifted towards larger particles. Weschler and Shields [53] carried out experiments with a limonene source placed in offices to investigate ozone influence on sub-micron particle formation initiated by terpene ozonolysis. Those results also indicated that the ozone concentration increase caused immediate particle number density growth. There are also several other reported studies on terpene-related particle formation in specific indoor environments, such as those concerning aromatherapy spa centers [51,54] and supermarkets [55], which confirmed as well the impact of terpenes’ oxidation on the formation of nano-sized particles.

#### 2.1.2. PTR-TOF-MS Experiments

PTR-TOF-MS experiments were conducted to compliment the results obtained by SMPS. PTR-TOF-MS with H_3_O^+^ soft ionization was characterized by low fragmentation of analytes. The calculation of VOCs concentration on the basis of the raw signal value (without calibration) is acceptable if the reaction rate coefficients *k* between given VOC and H3O^+^ ions and corresponding product branching ratios are known [56]. VOCs concentration can be calculated by the formula proposed by Lindinger and co-workers [57]:

(1) $$\left[\mathrm{VOC}\right]=\frac{1}{kt}\times \frac{\left[\mathrm{VOC}\cdot H^{+}\right]}{\left[H_{3}O^{+}\right]}$$

where: $\left[\mathrm{VOC}\cdot H^{+}\right]$—ion count rates for protonated VOC ions; $\left[H_{3}O^{+}\right]$—ion count rate for primary ion H_3_O^+^; *k*—reaction rate coefficient between VOC and H_3_O^+^; *t*—residence time of primary ions in the drift tube (typically 100µs)

There are several papers in which the authors pointed out the importance of several factors’ effects on the interference of a measurement, i.e., (i) the abundance of protonated water cluster ions [H_3_O(H_2_O)n]^+^, which depends on the PTR-MS working conditions and humidity of the sample (at “standard” operating conditions: RH 20–30%, ambient temperature 21 ± 1 °C, E/N range 120–140 Td, the ratio of the densities of H_3_O^+^H_2_O to H_3_O^+^ is usually less than 3%) [58,59]) and (ii) the possibility of the fragmentation of the compounds of interest (monoterpenes in this study) even under soft H_3_O^+^ ionization. In order to calculate VOCs’ concentration taking into account the contribution of all fragments, the following formula is used [56]:

(2) $$\left[\mathrm{VOC}\right]=\frac{1}{kt}\times \frac{\sqrt{{\left(m/z\right)}_{H_{3}O^{+}}}}{kt\;{\left[H_{3}O^{+}\right]}_{measured}}\underset{i}{\sum }\frac{{\left[VOC_{i}\cdot H^{+}\right]}_{measured}}{\sqrt{{\left(m/z\right)}_{VOC_{i}\cdot H^{+}}}}$$

where: $\left[VOC_{i}\cdot H^{+}\right]$—contribution from all fragments

The PTR-TOF-MS default reaction rate constant *k* applied to calculate the VOC concentration was equal to 2 × 10^−9^ cm^3^ molecule^−1^ s^−1^. However, for different VOCs, the *k* values may be slightly different, e.g., the experimentally determined *k* values for α-pinene and limonene were equal to 2.2 × 10^−9^ cm^3^ molecule^−1^ s^−1^ and 2.3 × 10^−9^ cm^3^ molecule^−1^ s^−1^, correspondingly [60]. The application of the default reaction rate constant for the determination of VOCs concentration may cause some over- or under-estimation of the calculated concentration, but the measured *k* values were generally within ±20% of the estimated rate constant [61], which should not cause significant errors. In this experiment, the default reaction rate constant was applied. The verification of PTR-TOF-MS concentration measurements, carried out with gaseous standard mixtures of (R)-(+)-limonene and α-pinene and the TD-GC-FID technique, revealed that the concentration measured by PTR-TOF-MS was burdened with an error in the range of 16–26% in all cases. Since the main goal of this study was to investigate the dynamism of monoterpene oxidation and the determination of the exact concentration values was not the priority, this error did not cause significant result bias.

In order to achieve the best balance between the fragmentation of the compounds of interest and the formation of water cluster ions, an E/N value equal to 103 Td was chosen for the experiments. For instance, the monoterpene concentration was determined mainly on the basis of the 137 *m*/*z* ion signal; therefore, it was crucial to choose an E/N value at which 137 *m*/*z* would be the ion of the highest signal intensity. The experiments carried out with the default E/N ratio of 122 Td revealed the decrease in 137 *m*/*z* ion signal intensity and the increase of the signal intensity of a main fragment ion 81 *m*/*z*.

Ions 137 *m*/*z* and 81 *m*/*z* were monitored in order to control monoterpenes’ concentration. Only the main ions of the oxidation products are presented in the graphs, and since oxidation products’ concentration changes were small and at various concentration levels, the results are presented in single graphs per each oxidation product ion (in order to make them clearer and easily visible). Other oxidation products’ fragment ions are mentioned in the text.

In the following part of this subsection, the results obtained by PTR-MS measurements of the concentration of the defined compounds introduced into the reaction chamber and formed in the ozonolysis reaction that took place there are given. The origin of the coordinate system in each graph is the point where ozone was introduced into the reaction chamber and, at the same time, the starting point of concentration changes monitored by PTR-MS. It should be pointed out here that apart from the products of monoterpenes’ oxidation monitored in this study, there are other (recently reported) compounds originating from monoterpenes’ oxidation, precisely autoxidation, such as s C_10_H_14_O_9_ or C_20_H_30_O_16_. These low-volatility vapors are named Highly Oxygenated Molecules (HOM) and are believed to contribute in a significant way to SOA formation by nucleation on pre-existing particles. Although the formation of HOM is referred to atmospheric conditions, it should not be neglected while considering the ultrafine particle-related processes occurring indoors, especially because many important aspects of HOM formation and the properties remain unknown [62]. Nevertheless, PTR-MS in the configuration used in this study is not able to measure HOM. In spite of the limitations of the PTR-MS system to characterize HOM, in the literature, one may find reported attempts with re-designed gas inlets (which significantly reduces wall losses) and reaction chambers (30 times longer reaction time and 40 times higher pressure in comparison to the standard PTR-TOF-MS configuration) to measure HOM, which ended up with satisfactory results [63,64].

**Figure 6:**
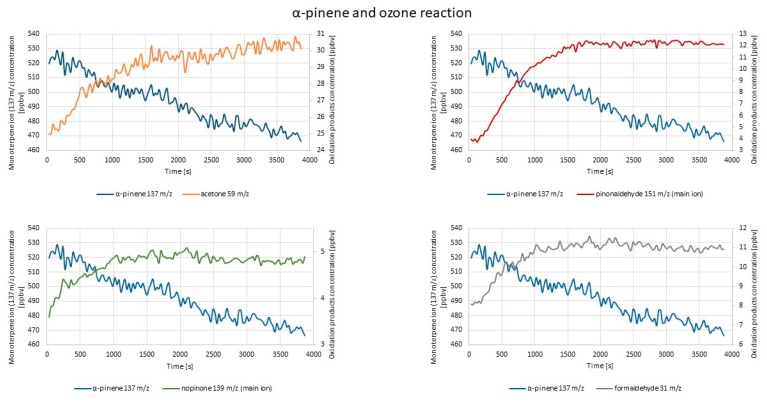
Exemplary results of PTR-TOF-MS concentration monitoring of oxidation products formed via α-pinene and O_3_ reaction.

Figure 6 represents exemplary results obtained during the measurement of the concentrations of the monitored compounds after α-pinene and ozone were introduced into the reaction chamber.

The highest concentration increase was observed in the case of the 151 *m*/*z* ion (pinonaldehyde fragment ion). The concentration increase occurred immediately after ozone introduction, but a significant rise was observable after 150 s, simultaneously with the monoterpene concentration decrease. Parent acetone (59 *m*/*z*) and formaldehyde (31 *m*/*z*) ions’ concentration also increased visibly. For remaining masses of pinonaldehyde fragment ions (109 *m*/*z*, 152 *m*/*z*) and the nopinone fragment ion (139 *m*/*z*), the concentration increase occurred almost immediately after ozone introduction; however, it was not so sharply visible. The nopinone fragment ion 93 *m*/*z* concentration changes in this experiment showed a similar (decreasing) trend as monoterpene ions (137 *m*/*z* and 81 *m*/*z*). According to another research work, the relative abundance of the mass (93 *m*/*z*) was very low as well [30]. These patterns may be explained by different scenarios: (i) 93 *m*/*z* was not a fragment ion of oxidation products, but a fragment ion of the monoterpene; (ii) further chemical reactions of oxidation products (nopinone and pinonaldehyde may undergo reactions that lead to acetone formation or reactions with OH radical [30]); (iii) condensation onto the formed particles (nopinone presence led to earlier nucleation in the system [65]).

**Figure 7:**
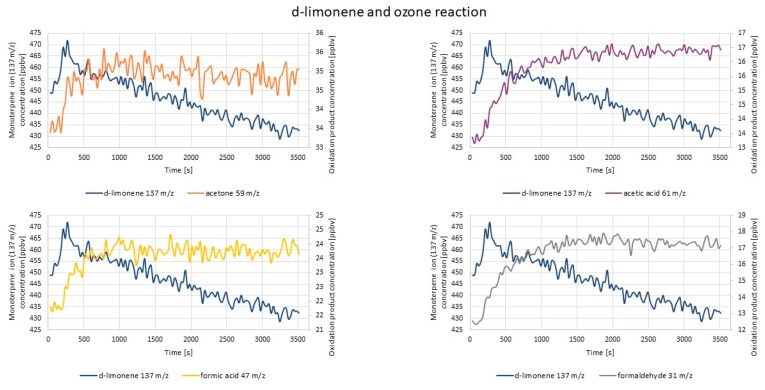
Exemplary results of PTR-TOF-MS concentration monitoring of oxidation products formed via d-limonene and O_3_ reaction.

Figure 7 represents the exemplary results of the PTR-TOF-MS measurements of the concentrations of the monitored compounds after d-limonene ozonolysis reaction.

The most visible concentration increase was in the case of formaldehyde (31 *m*/*z*), and it started to rise immediately after ozone introduction. Evident concentration increases were also observed for acetone and acetic and formic acids (59 *m*/*z*, 61 *m*/*z*, and 47 *m*/*z*, respectively). For limonaketone (139 *m*/*z*), a concentration increase also occurred immediately after ozone introduction. Subsequently, the concentration was rather constant, slightly decreasing at the end of the measurement. The increase of the concentration of 75 *m*/*z* (C_3_H_6_O_2_) and 155 *m*/*z* (C_9_H_14_O_2_) ions after ozone introduction was barely noticeable. The obtained results were in accordance with those reported in the literature [66].

**Figure 8:**
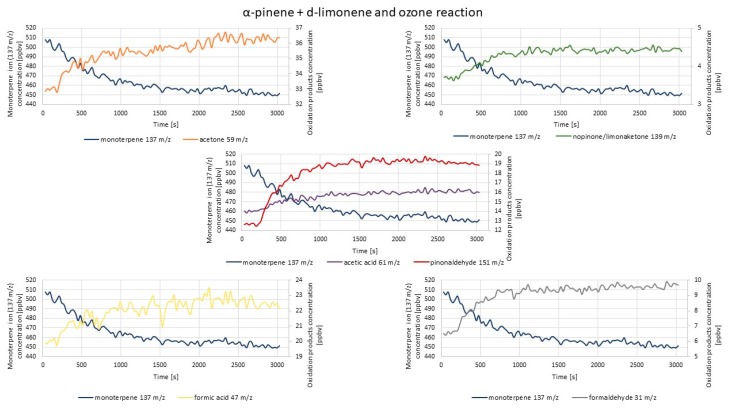
Exemplary results of PTR-TOF-MS concentration monitoring of oxidation products formed via α-pinene + d-limonene and O_3_ reaction.

Figure 8 represents exemplary results of PTR-TOF-MS concentration measurements conducted during the experiment aimed at investigation of whether the mixture of two monoterpenes would have any effect on the formation of the monitored oxidation products.

The trends described in the previous part of this section may be observed here as well. The most visible concentration increase was for pinonaldehyde ion 151 *m*/*z* and formaldehyde ion 31 *m*/*z* (58.3% and 66.7%, correspondingly). The concentration of ions 61 *m*/*z*, 59 *m*/*z*, and 47 *m*/*z* (acetic acid, acetone, and formic acid, respectively) also increased visibly during the measurement. The concentration increase of those ions lasted until the end of the measurement. One of the highest contributions observed in the case of pinonaldehyde ion 151 *m*/*z* might not have been expected, taking into consideration the difference between the reaction rates of α-pinene (86.6 × 10^−18^ cm^3^ molecules^−1^s^−1^) and d-limonene (200 × 10^−18^ cm^3^ molecules^−1^s^−1^) with ozone [9,67]. The higher abundance of products deriving from d-limonene ozonolysis was rather expected. This indicated that mixing two reactive components may change the anticipated (on the basis of the results obtained with these two components investigated separately) reaction products and their abundance. The primary products formed from different sources may change the course of the reactions, affecting indoor air chemistry. The nopinone fragment ion 93 *m*/*z* concentration was decreasing since the beginning of the measurement. For other monitored ions (109 *m*/*z*, 121 *m*/*z*, 139 *m*/*z*, 152 *m*/*z*, 155 *m*/*z*), concentration increases were hardly observable; moreover, the concentration of nopinone fragment ion 121 *m*/*z* showed a decreasing trend throughout the measurement. The possible explanations for the decreasing nopinone concentration pattern are included above in this section. The results obtained by the introduction of the mixture of two monoterpenes were in accordance with the results obtained by separate introduction of each monoterpene into a reaction chamber.

Summarized information on these results is presented in Table 2.

The greatest concentration variation was observable for 151 *m*/*z* ion, which according to the literature data [30], was the most abundant ion of pinonaldehyde, which was in accordance with the results of this study, despite different oxidants applied during the experiments. As reported in another study, the relatively high concentrations of the *m*/*z* 151 ion were also produced during α-pinene ozonolysis [68], but the authors defined this ion as deriving from verbenone, which is a possible α-pinene oxidation product created by OH radical oxidation [69]. However, most commonly, the 151 *m*/*z* ion is defined as deriving from pinonaldehyde [34,36]. Formaldehyde formation was also relatively dynamic in both experiments (monoterpenes alone and simultaneously introduced into the reaction chamber), which was in accordance with the results of reference studies [61,66]. Similar observation could be made in the case of acetone formation, which was not surprising, since acetone was mentioned as one of the major products created most commonly via α-pinene reactions with O_3_, OH, and NO_x_ [30,70,71,72]. On the other hand, in some reported studies on monoterpenes’ oxidation, acetone formation yields were rather low in the case of d-limonene as a substrate [5,61,73]. In the case of this study, despite the application of different oxidants than in reference studies, acetone formation was also greater during α-pinene ozonolysis than during d-limonene ozonolysis (however, taking into consideration the measurement uncertainty, the difference may be slight). Other ions deriving from compounds formed by α-pinene (121 *m*/*z*, 152 *m*/*z*, 93 *m*/*z*, 139 *m*/*z*, and 109 *m*/*z*) and d-limonene oxidation (75 *m*/*z*, 139 *m*/*z*, and 155 *m*/*z*) did not show significant concentration changes. The trend in alterations in ions 121 *m*/*z*, 152 *m*/*z*, 139 *m*/*z*, and 109 *m*/*z* concentrations was similar as reported in the literature [68]: firstly, slightly increased, which was followed by a steady decrease in time. Only in the case of 93 *m*/*z*, the concentration was constantly decreasing in time, both in this experiment and others reported in the literature [68]. The comparison of the changes in the concentration of less abundant ions corresponding to d-limonene ozonolysis products with other results of chamber studies reported in the literature [66] revealed both similarities and slight differences. For instance, the 75 *m*/*z* ion concentration measured in this study slightly increased after ozone introduction, and after 450 s, it was constant until the end of the measurement, whereas in the reference study [66], the 75 *m*/*z* ion concentration was increasing throughout the whole experiment. For the 155 *m*/*z* ion, the concentration change was barely visible in this study, as well as in reference. Differences in the results may be caused by the application of different experimental conditions in the reference study [66]. Moreover, the fact that oxidation products’ concentration became constant after some time in this study may be caused by the fact that there was no constant ozone supply during our experiments within the monitored time, so the substrate might have been consumed.

### 2.2. Experiments with Real Samples

The experiments with gaseous standard mixtures were extended with the investigation involving real monoterpene emission sources: Scots pines (known to be an abundant source of α-pinene [74]) and orange peel (as a source of limonene, which stands for 97% of the VOCs emitted from this product [75]). In this part of the study, pieces of Scots pine branch, shoots, and needles, as well as orange peel were separately placed in a micro-chamber connected to the reaction chamber, where the ozonolysis reaction was initiated (see the Materials and Methods section for details).

#### 2.2.1. SMPS Measurements

**Figure 9:**
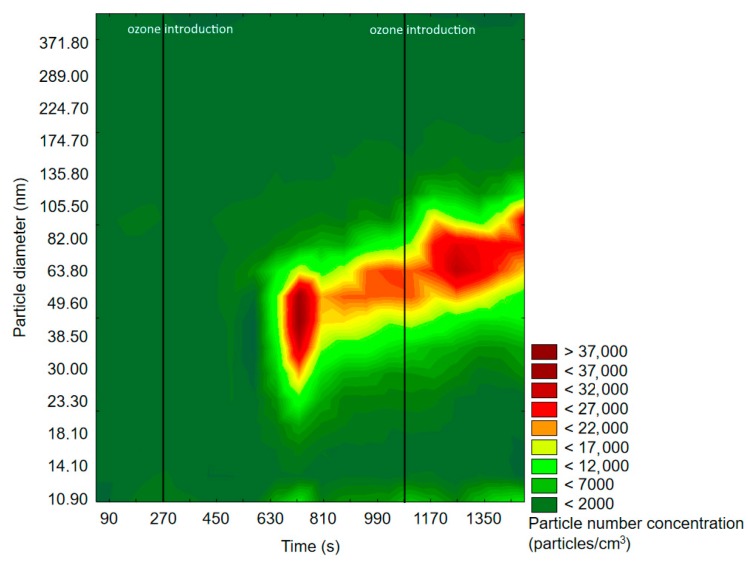
Exemplary results of SMPS measurement of particle size and space distribution changes initiated by Scots pine-emitted monoterpenes and O_3_ reaction.

**Figure 10:**
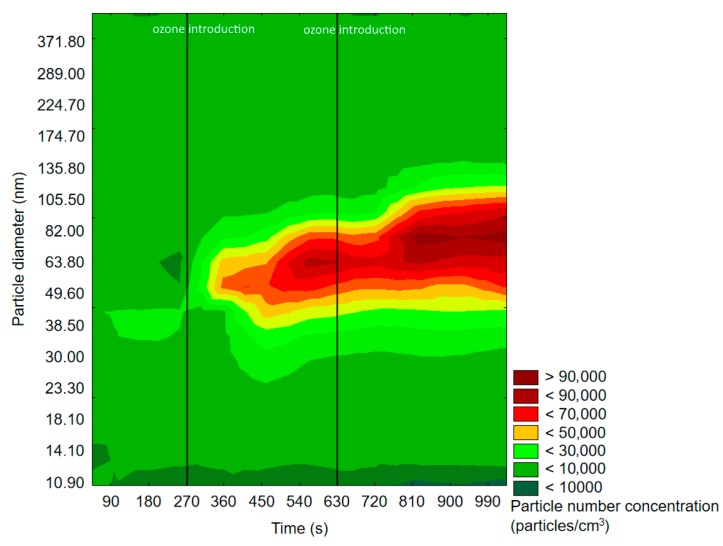
Exemplary results of SMPS measurement of particle size and space distribution changes initiated by orange-emitted monoterpenes and ozone reaction.

Exemplary results obtained by the SMPS measurements are presented in Figure 9 and Figure 10.

The measured maximum background PND after VOCs’ emission into the reaction chamber from Scots pine and orange peel was equal to 600 particles/cm^3^ and 12,200 particles/cm^3^, respectively. Each ozone introduction caused an increase in PND and a shift of PSD towards bigger diameters. The formation of particles initiated by ozonolysis process was observed after a significantly longer period of time (more measurement cycles) during the experiment with Scots pine in comparison to the one with orange peel. This difference was most likely related to the different reactivity of the components of orange and Scots pine essential oils. The terpene composition in Scots pine essential oil is diversified, and α-pinene is not a single dominating component there. It has been proven that branch emissions of Scots pine are rich in either 3-carene or pinenes (both α-pinene and β-pinene); however, the percentage share of these components depends on the trees’ chemodiversity. α-pinene and 3-carene together correspond to 40–97% of the monoterpene emission from Scots pine branch; however, a significant variability in this area has been pointed out, e.g., 10% of investigated tree samples emitted mainly α-pinene, and no 3-carene emission was reported, whereas in the case of 20% of the trees, 3-carene constituted over 80% of monoterpene emission measured. The average reported emission from sampled branches was characterized by an almost equal percentage share of α-pinene and 3-carene (ca., 40% each), 10% of β-pinene, and 10% of other compounds from monoterpene and sesquiterpene groups (e.g., limonene, camphene, terpinolene, cymene, β-caryophyllene, α-humulene) [76,77,78,79]. In contrast, d-limonene constituted up to 97% of the orange essential oil [75], but some trace levels of other terpenes (carvone, myrcene, sabinene, etc.), aldehydes, and alcohols were also present [80]. The reaction rates of the main (ca. 90%) Scots pine essential oil components with ozone (α-pinene 86.6 × 10^−18^ cm^3^ molecules^−1^s^−1^; 3-carene 37 × 10^−18^ cm^3^ molecules^−1^s^−1^; β-pinene 15 × 10^−18^ cm^3^ molecules^−1^s^−1^) were lower than the d-limonene/ozone reaction rate, which was equal to 200 × 10^−18^ cm^3^ molecules^−1^s^−1^ [9,67]; therefore, the SOA formation rate in the case of orange peel was higher. Moreover, the oxidation of the volatile fraction of orange peel seemed to produce more SOA particles than VOCs by Scots pine branch, which was consistent with the data available in the literature. It has been proven in many reported studies that limonene ozonolysis produces higher SOA levels than α-pinene ozonolysis, which is related to different partitioning of the primary and secondary products of reaction between α-pinene/d-limonene and ozone [81].

#### 2.2.2. PTR-TOF-MS Measurements

**Figure 11:**
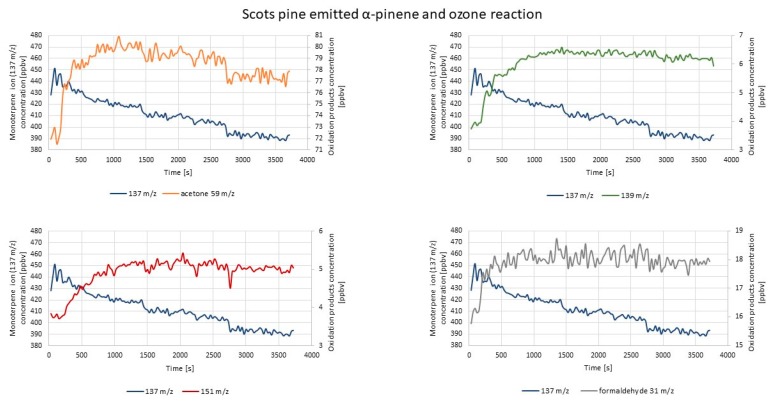
Exemplary results of PTR-TOF-MS concentration monitoring of oxidation products created via Scots pine-emitted monoterpenes and ozone reaction.

**Figure 12:**
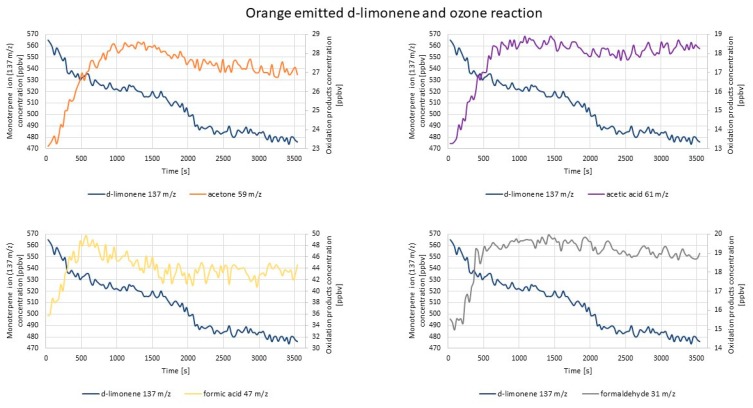
Exemplary results of PTR-TOF-MS concentration monitoring of oxidation products created via orange-emitted monoterpenes and ozone reaction.

Figure 11 and Figure 12 represent exemplary results obtained by PTR-TOF-MS measurements during the investigation on Scots pine-/orange peel-emitted monoterpenes and ozone reaction.

Although the α-pinene percentage share in Scots pine essential oil was rather abundant, it was not its dominant component (see the discussion in the previous section); therefore, in this case, ions 137 *m*/*z*, 139 *m*/*z*, and 151 *m*/*z* would not be denoted as α-pinene, nopinone, and pinonaldehyde (correspondingly) since there was the likelihood that they derived from other monoterpenes emitted from this source. Ozone introduction in this case caused the most rapid increase of acetone and formaldehyde concentration, whereas the concentration increase of the 139 *m*/*z* ion (possibly the nopinone parent ion) was the most significant (by 50% of its initial value). For the remaining ions 121 *m*/*z*, 152 *m*/*z*, 151 *m*/*z*, and 109 *m*/*z*, the concentration changes were barely visible, and the 109 *m*/*z* ion concentration decreased throughout the measurement. Such results differed from those obtained by studies involving α-pinene gaseous standard mixture, where the 151 *m*/*z* ion (pinonaldehyde fragment ion) concentration change was the most visible one. However, in accordance with this was the fact that the concentration changes of the ions of formaldehyde (31 *m*/*z*) and acetone (59 *m*/*z*) were clearly noticeable in both experiments. Differences between the results of ozonolysis of α-pinene and Scots pine-emitted terpenes were probably related to the diversified chemical composition of Scots pine (discussed in the previous section), which had implications on both particle formation initiated by the oxidation of essential oil components and chemical reactions occurring via the oxidation process. α-pinene was not a single dominating component in Scots pine essential oil; therefore, the abundance of oxidation products may be different in comparison to α-pinene oxidation products.

Since d-limonene constituted over 90% of orange essential oil, ions 137 *m*/*z* and 139 *m*/*z* may be assigned with a high amount of certainty to d-limonene and limonaketone, correspondingly. Ozone introduction caused the most visible concentration increase of the formic acid-derived ion (47 *m*/*z*). The limonaketone (139 *m*/*z*) concentration increase was also sudden after ozone introduction; later, it was constant and decreased at the end of measurement. The concentration changes of ions 75 *m*/*z* and 155 *m*/*z* were not so significant; however, the concentrations of those ions increased within 15 min of measurement and later remained constant until the end of it. The results were similar to those obtained in the case of experiments carried out with the d-limonene standard gaseous mixture, which was expected taking into consideration the dominance of d-limonene in orange essential oil. There were only small differences between the concentration levels of acetone and formic acid. In the case of the application of the d-limonene standard, acetone was a product of the highest concentration, while in the case of orange peel, it was formic acid. The reason for this slight difference most likely again lied in the minor contribution of other than d-limonene essential oil components (see the discussion in the previous section).

The summarized information on the results obtained within PTR experiments are presented in Table 3.

The experiments with real samples clearly indicated the significant contribution of these natural terpenes’ emission sources to SOA particles formation in indoor air, affecting in this way indoor air chemical composition. Orange peel and Scots pine branch represented different types of monoterpene emission sources indoors, meaning episodic and continuous sources. Episodic emission sources are characterized by high levels of emission in a short defined time of exposition; in this case, this would be the peeling of an orange. By this process, significant amounts of d-limonene may be introduced into the indoor air, increasing its concentration there up to tenfold [48]. Worth emphasizing is also the damaged structure of the emission source (orange peel), which affects the level of d-limonene emission indoors, unlike in the case of emission from unpeeled orange. Emission from Scots pine branch indoors may be assumed as a type of continuous emission, similar, e.g., to plug-in air fresheners (different from typical continuous sources indoors as for instance wooden furniture, but causing short-term continuous emission) and rather seasonal (e.g., Christmas trees that are present indoors in a defined period during a year). Experiments carried out with real samples proved that both episodic and continuous monoterpene emission sources significantly influenced the indoor air and therefore should be taken into account while investigating the specifics of indoor air with regard to SOA formation.

## 3. Materials and Methods

All of the experiments were carried out in a laboratory room; therefore, “ambient/background air” refers to the indoor air inside the laboratory room.

Chemical reagents applied in the study were:

- (R)-(+)-limonene 97% (Sigma Aldrich, St. Louis, Missouri, USA);
- (+)-α-pinene 98% (Sigma Aldrich, St. Louis, Missouri, USA);
- 1-butanol, EMSURE ACS (Merck, Darmstadt, Germany);
- Ozone was obtained from ozone generator GO 4-100 No 01, power supply voltage 220 V, power 50 VA.

Natural terpene emission sources applied in the study were:

- Orange fruit (peel);
- Scots pine (branch);

All experiments were conducted as follows: A crimp cap vial with a PTFE/red rubber septa filled with 0.5 cm^3^ of terpene (d-limonene or α-pinene) or pieces of real samples (orange peel, Scots pine) was placed in one of the micro-chambers of 114 cm^3^ in volume (Micro-Chamber/Thermal Extractor Markes^®^, UK, Wales, Llantrisant). The micro-chamber working temperature was 60 °C, whereas the output flow rate was 40 cm^3^ min^−1^. A constant stream of nitrogen (99.999%) flushed the micro-chambers for one hour, until the equilibrium state was reached, then an outlet of the micro-chamber was connected to an inlet of the reaction chamber (63 cm × 36 cm × 36 cm, made of polished stainless steel, inert to VOCs) to enrich the ambient air in the reaction chamber with terpenes or VOCs emitted from real samples. Once the required terpene concentration in the reaction chamber was reached, the inlets of SMPS and PTR-TOF-MS were connected to the reaction chamber, and the measurement was initiated. Ozone was introduced into the chamber with a gas-tight syringe through the inlet with PTFE/red rubber septa. The chamber was equipped with a mixing fan. The reaction chamber was ventilated after each experiment by opening the upper cover for 30 min. After this time, background measurement was carried out (for 10 min), and the concentrations of terpenes, formaldehyde, and acetone (main substrates and analytes controlled during experiments) were in the range of the concentrations of these compounds measured in the background air of the room.

RH was monitored with a hygrometer, and its value for the room background air was 35%, maintained by a working air conditioning system; therefore, inside the reaction chamber, the humidity was the same during all experiments.

### 3.1. SMPS Measurements

Measurements of the size distribution of nanoparticles were done by the application of the SMPS^TM^ TSI GmbH 3938L50 equipped with an impactor of 0.0508 cm in diameter, ^85^Kr neutralizer, Differential Mobility Analyzer (DMA) (long DMA, 44,369 cm, Model 3081A), and Condensation Particle Counter (CPC) (Model 3750). The impactor provided the cut off for the particles out of the range of measurement; the neutralizer was applied to dispose the charge of monitored particles, so they got only one, known type of charge, which reduced the electrostatic interactions between particles and the material the device was made of, whereas DMA arranged particles of different sizes according to their mobility (the specified range of voltage applied to the rod inside provided splitting and separating the particle beam of a defined size). Nanosized particles could not be detected by the usual optic detection system without the application of the condensation process, which provided an increase of the size of particles further counted by CPC. The working parameters during SMPS measurements were as follows: detector sample flow 1.0 dm^3^ min^−1^, sheath flow 5.0 dm^3^ min^−1^, aerosol flow 0.506 dm^3^ min^−1^, scan time 90 s, voltage range 10.75–9899.38 V, relative sample humidity 56–65%, sample temperature 23–26 °C. 1-butanol was used as the condensation fluid. The SMPS device was set in a working range to measure particles of a diameter ranging from 11.1 nm to 469.8 nm. SMPS software was provided by TSI, and for the device control program, AIM 2018 TSI (TSI, Shoreview, Minnesota, USA) was applied, whereas for data analysis, Aerosol Instrument Manager TSI Version 10.3 (TSI, Shoreview, Minnesota, USA) was applied. To create graphs representing particle size distribution changes, the program Statistica 13 (StatSoft Polska Sp. z o.o., Kraków, Poland) was applied.

### 3.2. PTR-TOF-MS Measurements

Products formed via the ozonolysis reaction were detected and quantified by PTR-TOF-MS 1000 Ultra Ionicon^®^ with H3O+ as primary ions. The principle of PTR-TOF-MS operation was described in detail elsewhere [82,83]. In brief, this technique applies soft H^3^O^+^ ionization that took place only if the proton affinity of an analyte was higher than the proton affinity of water (7.2 eV). Analytes could be determined based on the value of their mass plus the mass of a proton (*m*/*z*^+1^). The created ions were then separated and detected by high resolution TOF-MS. The working parameters during PTR-TOF-MS measurements were as follows: drift pressure 2.59 mbar, drift voltage 520 V, E/N 103 Td, drift temperature 70 °C, inlet temperature 70 °C, sampling velocity 1.5 cm^3^ min^−1^. In all experiments, the proton transfer reaction rate constant (k) of 2 × 10–9 cm^3^ molecule^−1^ s^−1^ was applied. PTR software was provided by Ionicon, and for device control, the program IoniTOF Version 3.0 (Ionicon Analytic, Innsbruck, Austria) was applied, whereas for data analysis, the program PTR-MS Viewer Version 3.3.8 (Ionicon Analytic, Innsbruck, Austria) was used.

The masses (protonated) selected to be monitored according to literature data [30,66,68] are listed below:

- α-pinene oxidation products: acetone (59 *m*/*z*) [30]; formaldehyde (31 *m*/*z*) [68]; nopinone (139 *m*/*z*, 140 *m*/*z*, 122 *m*/*z*, 121 *m*/*z*, 93 *m*/*z*, 83 *m*/*z*); pinonaldehyde (151 *m*/*z*, 170 *m*/*z*, 169 *m*/*z*, 152 *m*/*z*, 123 *m*/*z*, 109 *m*/*z*, 108 *m*/*z*, 107 *m*/*z*, 99 *m*/*z*, 72 *m*/*z*, 71 *m*/*z*, 43 *m*/*z*) [30]; pinonic acid (186 *m*/*z*); norpinonaldehyde (155 *m*/*z*) [68]; 10-OH-pinoninc acid (201 *m*/*z*) [68];
- d-limonene oxidation products: acetone (59 *m*/*z*); formaldehyde (31 *m*/*z*); formic acid (47 *m*/*z*); C_3_H_6_O_2_ (75 *m*/*z*); limonaketone (139 *m*/*z*); C_9_H_14_O_2_ (155 *m*/*z*); limononaldehyde (169 *m*/*z*); acetic acid (61 *m*/*z*); acetaldehyde (45 *m*/*z*) [66].

The concentration of all ions listed below was determined by measuring the ion count rates of the most abundant ions (according to the literature data). It is possible that some interference from the reaction products may result in the production of other ions with these masses (even if there was no evidence of interfering ions/compounds); therefore, the measured values should be considered as upper concentration limits. Moreover, the main goal of this research was to determine the dynamism of the terpene oxidation reaction, not the precise concentration. Experiments were designed in a way to minimize terpene fragmentation, and TD-GC-FID was additionally applied to monitor terpene concentration.

All measured ion signals were corrected for PTR transmission similarly as was done in other studies [82]. To set transmission factors, the Ionicon VOC MIX 2018 standard gaseous mixture was applied. The mixture contained 7 different VOCs with the following masses (*m*/*z*): 21 (hydronium ion), 42 (acetonitrile), 59 (acetone), 79 (benzene), 93 (toluene), 107 (ethylbenzene), 113 (chlorobenzene), and 147 (dichlorobenzene). PTR software was provided by Ionicon, and for device control, the program IoniTOF Version 3.0 (Ionicon Analytic, Innsbruck, Austria) was applied, whereas for data analysis, the program PTR-MS Viewer Version 3.3.8 (Ionicon Analytic, Innsbruck, Austria) was used.

### 3.3. TD-GC-FID Measurements

The determination of terpenes’ concentration in the background air and inside the chamber was carried out with the use of sorption tubes filled with Tenax TA^®^ 35/60 100 mg (recommended for trapping VOCs from air samples [84]), a thermal desorption unit (Markes^®^ Series 2), and a GC (Agilent Technologies 6890, MS Agilent Technologies 5973) and flame-ionization detector (GC-FID Agilent Technologies 7820A). The procedure was based on the methodology developed and applied in a different study published by the authors [85]. Before the analysis, sorbent tubes were conditioned in an inert gas atmosphere under 300 °C for 6 h using a thermal desorption unit (Markes^®^ Series 2). After conditioning, the tubes’ purity was verified by a blank chromatographic run (GC-FID Agilent Technologies 7820A) to confirm that the sorbent was free from impurities. Conditioned sorbent tubes were sealed using two-piece brass storage caps filled with one-piece PTFE ferrules (6 mm i.d.) and additionally closed in screw cap glass vials before analysis. To determine the concentration in the reaction chamber, 1.0 L of air was actively passed through the sorbent, then the tubes were sealed again and analyzed immediately. The thermal desorption parameters were as follows: 1 min (split ON) prepurge, 10 min desorption under 300 °C (split OFF), trapping analytes under 1 °C and release onto the chromatographic column under 300 °C (split OFF), trap heating time 5 min. The separation of the analytes was carried out on a DB1 column (30m × 0.32 mm × 5 µm, Agilent Technologies). The working parameters of the chromatographic system were as follows: column flow rate 2.2 mL min^−1^, temperature of TD-GC temperature program: 40 °C—1 min, 10 °C/min—125 °C, 15 °C/min—240 °C—5 min, detector temperature 250 °C. Data were evaluated by the OpenLab CDS ChemStation Workstation VL (Agilent, Santa Carla, California, USA).

## 4. Summary and Conclusions

The results obtained by SMPS and PTR-TOF-MS were in accordance with and complementary to each other. By the application of PTR-TOF-MS, it was possible to observe the increase of monoterpene ozonolysis products’ concentration (at the ppb level), the dynamism of the oxidation reaction changes, and to carry out the measurement in a real time. It was observed that firstly, the oxidation products were formed, and then, they underwent nucleation and condensation, forming particles whose diameter grew in time. At the very beginning, even a small increase in oxidation products’ concentration caused an increase in PND, which was rapid and reached high values. Firstly, small particles were formed in large amounts, then the maximum particle number density decreased, and the particle size distribution shifted towards particles of a bigger diameter, which proved that the nucleation and condensation processes took place. This phenomenon was also supported by the PTR-TOF-MS results, showing that initially, oxidation products’ concentration increased more rapidly, and with the passing of time, it became steady, which indicated particle formation. It was confirmed that ozone and terpene reactions led to the formation of oxidation products almost immediately after ozone introduction into the chamber containing terpenes. The formed oxidation products were different depending on the type of emission source applied. The significant concentration increases observed for formaldehyde and acetone were especially interesting, since it is generally known that those two compounds are emitted into the indoor air from various emission sources [4,86] and that they alone negatively influence indoor air quality [87,88]. It is worth emphasizing also the results obtained within the experiments with real samples commonly present and used indoors. Peeling an orange or placing Scots pine essential oil natural emission source (e.g., Christmas tree) indoors may have a substantial impact on indoor air chemistry and indirectly contribute to adverse health effects residents report as “sick building syndrome”.

Future directions in indoor air chemistry investigations concern a holistic approach of characterizing all the potential contributors to indoor air quality deterioration, i.e., human activity, reactive species emission sources, surface reservoirs, variability related to the specifics of a given indoor environment, low ventilation rates in new generation buildings, etc. The inclusion of highly oxygenated species into the compounds of interest should be also addressed. These are all aspects that may be explained most of all by the thorough investigation of processes that occur indoors, and therefore, this is worth further investigation.

## Figures and Tables

**Table 1:** Information about the experiments carried out with the SMPS instrument.

Experiment	Cycle of 1st O_3_ Introduction	Cycle of 2nd O_3_ Introduction	RH ^1^
α-pinene and O_3_ reaction	4th	11th	59%
d-limonene and O_3_ reaction	4th	11th	64%
α-pinene + d-limonene and O_3_ reaction	3rd	10th	64%
Scots pine wooden block emitted monoterpenes and O_3_ reaction	3rd	12th	59%
orange emitted monoterpenes and O_3_ reaction	3rd	7th	66%

^1^ Relative humidity.

**Table 2:** Summary of the results of PTR-TOF-MS experiments carried out with standard gaseous mixtures.

Experiment	Ion	Time Lag Between O_3_ Introduction and First Increase of the Oxidation Product Concentration (s)	Time Range while the Concentration of Oxidation Products Was Stable (s)	Trend at the End of the Measurement (increasing/decreasing/steady)
α-pinene ozonolysis	59	60	1740–3150	increasing
	151	30	1890–3840	steady
	139	30	1530–2340	decreasing
	31	30	1440–2040	decreasing
d-limonene ozonolysis	59	120	930–2190	decreasing/steady
	47	120	1290–3690	steady
	31	30	1290–2490	steady
	61	60	-	increasing
α-pinene + d-limonene ozonolysis	59	120	-	increasing
	47	120	-	decreasing
	31	120	990–2190	increasing
	61	150	-	increasing
	151	120	1590–2700	decreasing
	139	150	1290–3690	steady

**Table 3:** Summary of the results of PTR-TOF-MS experiments carried out with real samples.

Experiment	Ion	Time Lag Between O_3_ Introduction and the First Increase of the Oxidation Product Concentration (s)	Time Range while the Concentration of Oxidation Products Was Stable	Trend at the End of the Measurement (increasing/decreasing/steady)
Emission from Scots pine	59	30	-	decreasing
	151	60	1590–2640	decreasing/steady
	139	90	1590–3090	decreasing
	31	30	990–2790	decreasing
Emission from orange peel	59	30	-	increasing
	47	60	1830–3690	decreasing/steady
	31	90	2430–3690	steady
	61	30	-	increasing
